# Diabetic Foot: Current Status and Future Prospects

**DOI:** 10.1155/2016/5691305

**Published:** 2016-06-05

**Authors:** Didac Mauricio, Edward Jude, Alberto Piaggesi, Robert Frykberg

**Affiliations:** ^1^Department of Endocrinology and Nutrition, CIBER of Diabetes and Associated Metabolic Diseases, Health Sciences Research Institute and University Hospital Germans Trias i Pujol, 08916 Badalona, Spain; ^2^Diabetes Centre, Tameside Hospital NHS Foundation Trust, Ashton-under-Lyne OL6 9RW, UK; ^3^Manchester University and Manchester Metropolitan University, Manchester M13 9PL, UK; ^4^Sezione Dipartimentale Piede Diabetico, Dipartimento di Area Medica, Azienda Ospedaliero-Universitaria Pisana, 56124 Pisa, Italy; ^5^Department of Medicine, University of Pisa, Pisa, Italy; ^6^Phoenix VA Health Care System, Phoenix, AZ 85012, USA; ^7^Midwestern University, Glendale, AZ, USA

Diabetic foot disease is a debilitating complication of diabetes mellitus, ultimately affecting up to 50% of patients with both type 1 and 2 diabetes. Currently, this complication is still leading to significant loss of quality and years of life of the affected patient [1, 2]. Furthermore, it represents at least 12–15% of the overall cost associated with diabetes and up to 40% in developing countries [2, 3]. Additionally, current available treatments for diabetic foot disease are usually not as effective as they should be [4]. This is mainly explained by the insufficient knowledge of its underlying mechanisms and treatment tools because of the insufficient interest and research resources allocated to the study of this complication worldwide.

All, except one, of the papers included in this special issue are dealing with clinical and epidemiological aspects of diabetic foot disease with an observational design. One paper is devoted to the investigation of a novel therapeutic approach in an experimental animal model. The content of this issue mirrors the current clinical and research background of this diabetic complication; that is, there is ample room for improvement in all aspects of its prevention and treatment.

In one of the papers, using a retrospective study design, N. K. Chammas et al. assessed mortality and its causes in a large sample of patients from a single foot clinic in the UK and confirmed that death occurred 5 years earlier in patients with a diabetic foot ulcer. As for the general diabetic population, cardiovascular mortality was the main cause of death in the cohort, and the same was observed in patients with neuropathic foot ulcers where ischemic heart disease was the main cause of death. As found in previous studies, these results confirm the close link between diabetic microangiopathic disease and atherosclerotic cardiovascular disease. As for retinopathy and diabetic kidney disease, the results point again to neuropathic foot disease as a cardiovascular risk condition.

In a very large cohort of patients from Japan, a retrospective study by M. Tomita et al. aimed at assessing the incidence of diabetic foot ulcers according to the presence and severity of microvascular disease, that is, retinopathy and albuminuria. During a long follow-up period, after adjusting for other potential major contributing factors, the authors showed that the presence of concomitant retinopathy and albuminuria greatly increased the risk of developing diabetic foot disease. Thus, these findings should be taken into account in terms of preventative measures in patients with diabetic microangiopathic complications.

In the field of epidemiology, C. I. Bondor et al. performed a post hoc analysis using the data from a previous study of quality of life in a cross-sectional survey done in Romania in 2012. Although the study was based on self-reported data, they showed a very high frequency of foot ulcers and amputations in this large cohort of more than 21,000 diabetic patients. These results confirm the great burden created by diabetic foot disease and highlight the importance of epidemiological studies to raise the awareness of this important health care problem around the globe.

M. T. V. Quilici et al. performed a cross-sectional study in patients admitted to a Brazilian hospital because of diabetic foot infections. The findings of the study showed a high rate of amputations in this study cohort in which most patients had neuroischemic feet. Additionally, the study underlines the importance of early referral of patients with diabetic foot infections.

An interesting paper by M. May et al. showed the data on amputations from a large insurance company delivering health care to some 2.9 million people from 2 regions in Germany before and after the implementation of 6 networks for specialized care of the diabetic foot. They found that there was a decrease of overall amputations. However, the amputation rates were still high and indicate the need for further improvement in specialized care of the diabetic foot.

Finally, in the field of experimental research, X. Zhang et al. conducted an experimental study using Tuo-Li-Xiao-Du-San, a traditional Chinese medicine formula, to treat wounds in a rat model of diabetes. They were able to show that this treatment was able to enhance the healing of ulcers through several mechanisms: reduction of inflammatory cell mediators and increase in angiogenesis and collagen deposition.

To conclude, although the interest in research and management of diabetic foot disease has increased in recent years, it is still the Cinderella among diabetes complications in terms of research efforts and in resource allocation and outcomes. Additionally, the complexity of its management needs to be addressed on a multidisciplinary approach that should gather all expertise necessary for the optimal management of each aspect of this complication [2, 5]. The future should bring much more high-quality research to the area of diabetic foot disease, from basic research to properly conducted clinical trials.
